# Human Immunodeficiency Virus (HIV) types Western blot (WB) band profiles as potential surrogate markers of HIV disease progression and predictors of vertical transmission in a cohort of infected but antiretroviral therapy naïve pregnant women in Harare, Zimbabwe

**DOI:** 10.1186/1471-2334-11-7

**Published:** 2011-01-06

**Authors:** Kerina Duri, Fredrik Müller, Felicity Z Gumbo, Nyaradzai E Kurewa, Simba Rusakaniko, Mike Z Chirenje, Munyaradzi P Mapingure, Babill Stray-Pedersen

**Affiliations:** 1 Department of Immunology, University of Zimbabwe, Harare, Zimbabwe; 2 Institute of Microbiology, Rikshospitalet Oslo University Hospital and University of Oslo, Oslo, Norway; 3 Department of Paediatrics and Child Health, University of Zimbabwe, Harare, Zimbabwe; 4 Division of Women and Children, Rikshospitalet Oslo University Hospital and University of Oslo, Oslo, Norway; 5 Department of Community Medicine, University of Zimbabwe, Harare, Zimbabwe; 6 Department of Obstetrics and Gynecology, University of Zimbabwe, Harare, Zimbabwe

## Abstract

**Background:**

Expensive CD4 count and viral load tests have failed the intended objective of enabling access to HIV therapy in poor resource settings. It is imperative to develop simple, affordable and non-subjective disease monitoring tools to complement clinical staging efforts of inexperienced health personnel currently manning most healthcare centres because of brain drain. Besides accurately predicting HIV infection, sequential appearance of specific bands of WB test offers a window of opportunity to develop a less subjective tool for monitoring disease progression.

**Methods:**

HIV type characterization was done in a cohort of infected pregnant women at 36 gestational weeks using WB test. Student-t test was used to determine maternal differences in mean full blood counts and viral load of mothers with and those without HIV *gag *antigen bands. Pearson Chi-square test was used to assess differences in lack of bands appearance with vertical transmission and lymphadenopathy.

**Results:**

Among the 64 HIV infected pregnant women, 98.4% had pure HIV-1 infection and one woman (1.7%) had dual HIV-1/HIV-2 infections. Absence of HIV pol antigen bands was associated with acute infection, p = 0.002. All women with chronic HIV-1 infection had antibody reactivity to both the HIV-1 envelope and polymerase antigens. However, antibody reactivity to gag antigens varied among the women, being 100%, 90%, 70% and 63% for p24, p17, p39 and p55, respectively. Lack of antibody reactivity to gag p39 antigen was associated with disease progression as confirmed by the presence of lymphadenopathy, anemia, higher viral load, p = 0.010, 0.025 and 0.016, respectively. Although not statistically significant, women with p39 band missing were 1.4 times more likely to transmit HIV-1 to their infants.

**Conclusion:**

Absence of antibody reactivity to pol and gag p39 antigens was associated with acute infection and disease progression, respectively. Apart from its use in HIV disease diagnosis, WB test could also be used in conjunction with simpler tests like full blood counts and patient clinical assessment as a relatively cheaper disease monitoring tool required prior to accessing antiretroviral therapy for poor resource settings. However, there is also need to factor in the role of host-parasite genetics and interactions in disease progression.

## Background

Acquired Immunodeficiency Syndrome (AIDS) is currently one of the most devastating diseases caused by HIV. Globally, in 2007 alone, 33 million people were living with HIV/AIDS and 20 million had died [1]. Studies have shown a cross-species transmission of HIV from a primate lentivirus to humans and the virus can be phylogenetically classified into two types; 1 and 2 [2]. This distinction is essential for accurate surveillance and diagnosis as well as administration of appropriate antiretroviral therapies within a population.

HIV type 1 (HIV-1) is the first in the class of human retroviruses and accounts for more than 95% of the world's HIV infections. Its origin can be traced back to a Simian Immunodeficiency Virus (SIV) isolated from a Chimpanzee (cpz) sub-species, *Pan troglodytes troglodytes *(SIVcpz)[3]. Both HIV-1 and SIVcpz have a unique *Vpu *gene in their respective genomic structures [2]. HIV-2 is the second in the same class and is largely confined to West Africa. Its closest relative is a monkey, sooty mangabey (sm), *Cercocebus atys*, SIVsm. A unique *Vpx *gene characterises both viruses' gene structures [4,5]. However, HIV-2 and HIV-1/HIV2 co-infections have also been documented outside West Africa [6]. HIV-1 and HIV-2 are closely related viruses with nucleotide sequence homology of 58%, 59% and 39% in the group specific antigen (*Gag), Pol *and *Env *genes encoding the viral nucleocapsid, polymerase enzymes and envelope glycoproteins, respectively [7]. Relative to HIV-1, HIV-2 has a reduced rate of transmissibility, disease development and has shown natural resistance to readily available non-nucleoside reverse-transcriptase inhibitors [8,9].

Classical algorithm of laboratory diagnosis of HIV infection has been the detection of anti-HIV antibodies using rapid tests with WB immunoassay as the gold standard method for validating screening test results [10,11]. However, in Zimbabwe, a large proportion of HIV diagnoses are currently being done without WB confirmation yet its banding profiles can yield valuable patient information. In this setting, WB test is only used as a tie-breaker in cases of discrepancy in the results. Unlike screening tests that detect antibodies to one or all HIV antigen(s) without specifying which antigen reacts to which antibody, the WB test with separated viral proteins immobilized on a membrane, generates specific information on the reactivity of patient antibodies to specific HIV antigens. Positive reactions appear as bands of numerous patterns [12]. Variations in WB band intensities, numbers, or their sequential order of appearance during different stages of HIV infection have been observed [13]. Following sero-conversion, anti-gag antibodies to p17, p24 and its precursor p55 appear first and tend to decrease with the onset of clinical symptoms [14]. A reduced prevalence of core antibodies has also been shown to be associated with the development of immunodeficiency [15]. In contrast, antibodies to *env *antigens have been detected in virtually all HIV infected persons regardless of clinical stage [16]. This sequential appearance of specific WB bands offers a window of opportunity to develop a simple and non-subjective disease assessment tool and also to predict the likelihood of vertical transmission.

High cost of CD4 count and viral load tests has hampered the intended objective of accessing HIV therapy in poor resource settings. Hence, there is a need for alternative initiative towards development of simple, accurate, affordable and non subjective disease monitoring tools. In view of the current brain drain challenge, this development would complement clinical staging efforts of inexperienced health personnel currently manning most healthcare centres.

WB test has been in use in Zimbabwe for some time now, mainly for HIV diagnosis. However, critical analysis of the band profiles regarding their additional potential applications has been overlooked. This study aimed to characterize HIV types among pregnant women using the WB test and to determine whether the presence or absence of particular band(s) correlated with HIV-1 disease progression or predicted vertical transmission.

## Methods

### Study Design and Setting

**Figure 1:**
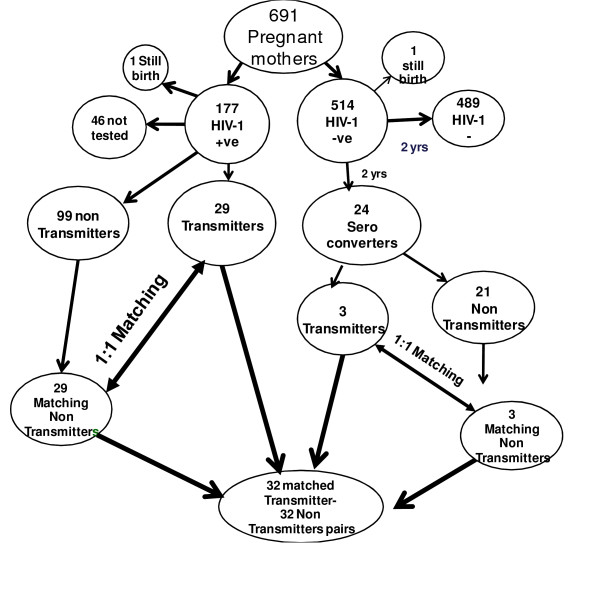
**Summary of how the 32 Transmitters and 32 non-Transmitters were sampled from a cohort of pregnant mothers attending antenatal clinics around Harare**.

This was a nested case-control study in which the cases and controls were sampled from a cohort of pregnant women attending 3 antenatal clinics around the city of Harare, Zimbabwe. All participants were part of a national **P**revention of **M**other-**T**o-**C**hild **T**ransmission (PMTCT) program and were naïve to antiretroviral therapy. The primary end point was an HIV-1 positive mother who transmitted the virus to her infant, transmitting mother (case). Each case was matched to one HIV-1 positive but non-transmitting mother (control). Matching of cases and controls was done with respect to important risk factors of HIV disease progression and vertical transmission notably maternal age, baseline sexually transmitted infections (STIs), clinical signs, the date of last menstruation and single dose nevirapine therapy, see figure 1.

### Study Population and Procedures

Pregnant women were enrolled at 36 gestational weeks between April and September 2002. Pre-and post-HIV test counseling services were readily available. HIV-1 positive mother and infant pairs were offered 200 mg single dose nevirapine during labour and 2 mg/kg body weight within 72 hours post delivery, respectively. Mothers were encouraged to exclusively breastfeed during the first six months post-delivery.

The study population consisted of two groups of pregnant HIV-1 positive women. The main group consisted of pregnant women who were HIV-1 positive at enrolment, considered to be having chronic HIV-1 infection, and a subgroup of pregnant women who were HIV-1 negative during pregnancy but sero-converted after delivery, thus regarded as having acute HIV-1 infection. Follow-up of HIV-1 negative mothers together with HIV-1 exposed infants was from delivery, 6 weeks, 4 and 9 months and thereafter 3 monthly until 2 years, thus generally coinciding with infant immunization visits. At each subsequent follow-up visit, HIV-1 negative mothers and exposed infants were re-tested for HIV-1 antibodies and antigens, respectively. Besides HIV testing, serum samples of sero-negative mothers and their respective infants were aliquoted and stored for further analysis.

### Mothers' and Infants' Demographic characteristics, Examination and sample collection

At enrolment all mothers answered a structured questionnaire and information regarding their socio-demographics, sexual behavioural, obstetric and reproductive health issues was obtained. A gynecologist performed physical and gynecological examinations.

A pediatrician examined infants. Date of birth, birth weight, gender and single dose nevirapine therapy were recorded. Five milliliters of maternal venous blood samples were collected in EDTA tubes at baseline and each follow-up visit in the cases of HIV-1 negative mothers. Two milliliters of venous EDTA whole blood samples were collected at each follow-up visit for HIV-1 negative but HIV-1 exposed infants. Samples were stored at -86°C until tested.

### Mothers' Tests

Serial HIV-1/-2 algorithm antibody tests were performed on plasma samples using Determine (Abbott Diagnostics, Illinois USA) and Ora-Quick (Abbott Diagnostics, Illinois, USA) rapid kits. Confirmation of screening HIV-1/2 rapid test results was done at the Norwegian Institute of Public Health using the WB test (HIV blot 2.2, MP Diagnostics, Singapore) according to the manufacturer's instructions. Interpretation of the WB test results was done in line with the World Health Organization guidelines [17]. A WB test was considered positive if at least two of the three envelope antigen bands for HIV-1 or glycoprotein (gp) 36 for HIV-2 and any of the four *gag *antigens or at least any one of the three *pol *antigens were present. A WB test result was considered to show dual reactivity when sera reacted with at least two *env *glyco-proteins and one core protein of each virus. Specimens with reactive gp36 antigen were re-run on a WB test specific to HIV-2.

Full blood counts were done using Abbott Diagnostic Cell Dyne 3500R SL Hematology Analyser. Plasma samples were shipped on dry ice to the Institute of Microbiology in Oslo to be quantified for HIV-1 RNA load using an automated TaqMan Roche Amplicor 1.5 Monitor Test (Cobas AmpliPrep/Cobas TaqMan, Roche Diagnostics, Branchburg NJ) according to the manufacturer's instructions as previously described [18]. The first available HIV-1 positive sample was quantified in the cases of sero-converters.

### Infants' Test

Detection of infants' HIV-1 infections was performed using qualitative 1.5 Roche Amplicor HIV-1 DNA PCR kit (Roche Diagnostics). Since this was a breastfeeding population, the criteria used to determine time of infection was similar to that used by Bertolli *et al. *[19]. Infants that tested HIV-1 DNA PCR positive on whole blood collected within 10 days of birth were considered to be infected *in utero*. Infants who had negative HIV-1 DNA PCR results within the first 10 days of life but had positive results at six weeks were regarded as infected during intra-partum and those testing positive thereafter were considered infected after birth.

#### Statistical Analysis

Data were entered and analyzed using STATA version 10. The frequency of WB bands were determined among the pregnant women in general and also after stratifying by the time of HIV infection (acute or chronic) and vertical transmission, as transmitting or non-transmitting mothers. A graph was plotted to show the frequency of different WB gag antigen bands between the two groups of mothers. Student-t test was used to determine differences in mean viral load and maternal hemoglobin between mothers with and those without *gag *antigen bands. Pearson Chi-square test was used to assess differences in the absence HIV *gag *antigen bands with vertical transmission and lymphadenopathy. Comparisons of the appearance of the HIV env, pol and *gag *antigens band profiles of mothers with chronic and those with acute HIV-1 infections were also done. Tests of statistical significance included the 95% confidence intervals of unadjusted relative risks and p values of less than 0.05 were considered statistically significant.

#### Ethical Consideration

The study was approved by the Medical Research Council of Zimbabwe and the Ethical Review Committee of Norway. Written consent to participate in the research study was obtained from the mothers and they were free to discontinue at any given time without any prejudice.

## Results

### Demography and Reproductive Health Characteristics of the 58 mothers: 29 transmitting and their 29 matched non-transmitting mothers with chronic HIV infection

Mean age (standard deviation) of the women was 26.6 (5.2) years, being 26.3 (5.6) and 25.6 (5.6) years for transmitters and non-transmitters respectively, p = 0.610.

All the women had at least 7 years in school and were not formally employed. Ninety-three percent were married and 90% had at least one child. All the mothers had spontaneous vaginal deliveries and were generally asymptomatic for HIV infection at enrolment.

There were two stillbirths, one among the HIV-1 positive and the other within the HIV negative group. These two were excluded from analysis. From the 176 HIV-1 positive mothers that delivered live births 126 (72%) mother-baby pairs were successfully followed up and tested. There were no differences with respect to socio-demographic characteristics, sexual behavior, reproductive genital tract infections and medical history between the 58 mothers with chronic HIV-1 infection constituting the main group in this study population and the rest of the mothers were HIV-1 positive at enrolment but were excluded or lost to follow-up. However, these 58 mothers were more likely to have more children, p = 0.016.

### HIV Prevalence, Types and Vertical Transmission of the 29 Transmitting and their Matched 29 Non-Transmitting Mothers with Chronic Infection

At baseline 691 pregnant women were enrolled of whom 177 (25.6%) and 514 (74.4%) were HIV-1 sero-positive and sero-negative, respectively. Performance concordance of the two serial HIV-1 rapid test results was 100%. Confirmatory WB tests of the 58 women with chronic HIV infection showed a 98.3% pure HIV-1 infection. None was found with solely HIV-2 infection. Only one woman (1.7%) had dual HIV-1/HIV-2 infections.

Twenty nine (23%) mothers transmitted the virus to their infants 10 (34%) and 19 (66%) during *in utero *and intra-partum/postpartum transmissions respectively.

### HIV Incidence, Type(s) and vertical transmission among 6 sero-converters: 3 transmitting and 3 non-transmitting mothers

Out of the 512 HIV-1 negative mothers that delivered live births, 24 sero-converted during the two year follow-up period, giving an HIV-1 incidence rate of 2.3 per hundred women years. Eighty-five percent of the mothers sero-converted after weaning their infants from breast-milk. Mothers with acute HIV-1 infections were generally younger relative to HIV-1 negative mothers in the cohort, with mean ages of 21.8 (4.6) and 23.7(5) years respectively, p = 0.06. More so, sero-converters were generally younger compared with mothers having chronic HIV-1 infection, mean (SD) ages, 21.8(4.6) and 26(5.5) years respectively, p = 0.04. There were no differences with respect to level of education, age of sexual debut, reproductive tract infections and STIs between the mothers with acute and those with chronic HIV-1 infections.

Among the 24 sero-converters with acute HIV-1 infection, three (13%) transmitted the virus to their infants through breastfeeding around 9 months postpartum. All the three infants were exposed, through breast milk for about three months before acquiring HIV-1 infection. All the sero-converting mothers had solely HIV-1 infection. Neither HIV-2 nor HIV-1/HIV-2 co-infections were detected in this subgroup.

### Frequency of HIV-1 WB Bands among 58 mothers with chronic HIV infection

Reactivity to all the 10 WB HIV-1 proteins was observed in 78% of the HIV-1 positive women. All specimens showed a strong positive reaction to both the HIV-1 envelope glycoproteins (gp160, gp120 and gp41) and the polymerase antigens (p31, p51 and p66). However, antibody reactivity to the gag core antigens varied among the women, being 100%, 90%, 70% and 63% with the p24, p17, p39 and p55 respectively. Absence of maternal antibody reactivity to HIV-1 *gag *demonstrated no relationship with maternal age, marital status, age of sexual debut, the number of sexual partners the women had had, current nor history of STIs.

**Figure 2:**
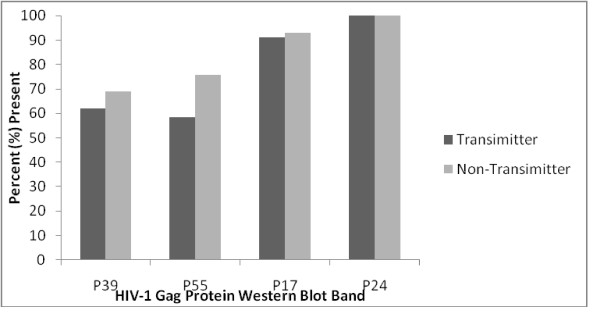
Frequencies of different gag protein among transmitters and non-transmitters

*Gag *p39 and p55 antigens were the most commonly missing bands among transmitting mothers. Generally band appearance was not significantly different when compared with band profiles of the non-transmitting mothers, see figure 2. Mothers who had *gag *p39 antigen bands missing were 1.4 times more likely to transmit the virus to their infants compared to those who had this band present though their number was too small to reach statistical significance, p = 0.104.

Lack of antibody reactivity to *gag *p39 antigen was significantly associated with disease progression as demonstrated by the presence of maternal lymphadenopathy, anaemia and higher viral load, p = 0.010, 0.025 and 0.016 respectively, see table 1. Women with p39 *gag *antigen band missing were about 6 times more likely to have a viral load of more than 10 000 copies/mL relative to their counterparts who had that band present, 5.58 [1.74-17.86].

**Table 1:** Associations of antenatal surrogate markers of disease progression of 58 pregnant women (chronic HIV-1 infections) with presence or absence of gag proteins reactivities

Antenatal variable	HIV-1 WB gag antigen band reactivity			
	Band	P17	P39	P55
**Lymphadenopathy Number/Total (%)**	Present	4/51 (8%)	0/32 (0%)	2/38 (5%)
	Absent	0/2 (0%)	4/21 (19%)	3/15 (13%)
	p-value	0.680	0.010	0.316
**Hemoglobin Mean (SD)**	Present	10.8 (1.3) N = 54	11.0 (1.2); N = 33	10.9 (1.4); N = 39
	Absent	11.7 (1.0) N = 2	10.4 (1.5); N = 23	10.6 (1.0); N = 17
	p-Value	0.312	0.025	0.395
**Viral load log**_**10 **_**Mean (SD)**	Present	3.3(1.0); N = 56	3.0 (0.8); N = 35	3.1 (0.9); N = 41
	Absent	2.3 (0); N = 2	3.7 (1.2); N = 23	3.6 (1.2); N = 17
	P-value	0.167	0.016	0.072
**Vertical Transmission**	Present	29/56 (52%)	15/36 (42%)	20/41 (49%)
**Number/Total (%)**	Absent	0/2 (0%)	14/22 (64%)	9/17 (53%)
	P-value	0.150	0.104	0.773

Presence of *gag *p17 antigen band was associated with established or advanced HIV-1 infection in mothers with chronic HIV-1 infection p = 0.002, see table 2.

**Table 2:** Reactivity of different env, pol and gag proteins of mothers with chronic HIV-1 infection and sero-converters

Positive HIV-1 antigen Band	Chronic infections	Sero-converters	p-value
	N = 58 (%)	N = 6 (%)	
***gag***			
**p17**	57 (98)	3 (50)	0.002
**p24**	57 (98)	5 (83)	0.183
**p39**	35 (60)	2 (33)	0.186
**p55**	41 (71)	2 (33)	0.251
***pol***			
**p31**	58 (100)	3 (50)	0.001
**p51**	58 (100)	4 (67)	0.002
**p66**	58 (100)	4 (67)	0.002
***Env***			
**Gp41**	58 (100)	6(100)	-
**Gp120**	58(100)	6(100)	-
**Gp160**	58(100)	6(100)	-

**Key**: P: protein, **g**p: glycoprotein

### Frequency of HIV WB Bands among mothers with acute HIV-1 infection

All the mothers with acute HIV-1 infection had antibody reactivity to all the 3 *env *antigens, gp41, 120 and 160. However, reactivity to HIV-1 *gag *antigens varied, but because of the small number of women in this subgroup, it was difficult to make any conclusive remarks. Lack of antibody reactivity to *pol *antigens, p31, p51 or p66 was significantly associated with early infection, hence absence of these bands on a WB test result could predict acute HIV-1 infection, see table 2.

#### WB band Profiles of the mother with HIV-1/-2 co-infection

In addition to the weak HIV-2 specific p36 band, reactivity to all the *env, pol *and *gag *antigens was observed. The mother was generally well with a relatively low baseline viral load of 690 viral copies per mL and did not transmit the viruses to her baby.

## Discussion

This study is a first attempt to correlate simple WB band profiles with disease progression and to some extent vertical transmission in Zimbabwe. The WB test is simple and easily interpreted by skilled users. Nevertheless, rapid tests are even simpler and can be conducted in rural settings without electricity. Although WB test remains relatively more expensive compared to rapid tests, it could be worthwhile using provided it yields a wealth of information on patients' serology. Besides accurately predicting HIV infection, sequential appearance of specific bands on WB test result offers a window of opportunity to develop a less subjective tool to monitor disease progression.

In our study of pregnant women recruited around Harare, HIV 1 was the predominant type with only one HIV-1/HIV-2 co-infected mother. This observation is in agreement with a previous bigger study, coincidentally also done in Harare in the same population that showed an HIV-2 and HIV-1/HIV-2 prevalence of 1.3% and 0.5%, respectively [20].

A recent study has shown that serological cross-reactivity for HIV-2 in HIV-1 infected individuals is rare when using synthetic peptide based assays which was the case with the MP Diagnostic HIV BLOT 2.2 kit we used in our study [21]. Similar to the findings of the same study, a weak reactivity to gp36 band was observed in the co-infected mother. However, a more specific HIV-2 PCR should have been done to confirm the HIV-2 immuno-blot test result. With the world fast becoming a global village, the possibility of importing and/or exporting new HIV types is inevitable, more so for most unemployed Zimbabweans who have resorted to cross-border trading with regional countries such as Mozambique and Angola with HIV-2.

Antibodies to the *env and pol *antigens were well detected in all the HIV infected pregnant women. Similar findings of band reactivities were reported earlier [13,16,22,23]. These results emphasise the importance of considering both the *env *and *pol *antigens in the interpretation criteria of WB HIV positive test results at least in this population. Antibodies to gag antigens, p17, p39 and p55 were not expressed efficiently in these women as was the case with other previous related studies [22,23]. Unlike the Indian study, where p55 antigen band was not detected at all in the patients with WHO clinical stage 1, our study observed a 63% expression of this antigen [22]. Interestingly in our study population, this antigen was the least expressed of all the WB test HIV antigens. This difference could be due to the fact that most of our women had surpassed the WHO clinical stage 1.

Fiebig *et al.*, have classified primary infection of HIV into seven stages incorporating WB test results. A characteristic band appearance indicative of an indeterminate test result has been shown to occur at stage IV with a true positive WB test result at stage V but without the p31 antigen band which only appears in the final acute infection stage VI [24]. Also observed in our study amongst sero-converters, was the absence of reactivity to *pol *antigens hence these could be predictors of sero-conversion. Analogous results have also been demonstrated by Sudha *et al.*, who have shown p31 antigen to be a predictor for early HIV infection [13]. Presence of p17 antigen band was associated with chronic infections and hence could be a predictor of established HIV infection contrary to the results observed by some studies [14,15]. Lack of antibody reactivity to p39 and p55 antigens was associated with disease progression as confirmed by the presence of lymphadenopathy, anemia, high viral load of above 10 000 viral RNA copies per mL and a higher likelihood of vertical transmission. Similar findings were also obtained elsewhere [25].

Without any intervention, majority HIV-1 positive pregnant women do not transmit the virus to their infants. Highly active antiretroviral therapy (HAART) is not yet readily available in PMTCT programs in resource limited settings. The long term side effect of single dose nevirapine therapy offered to most HIV positive pregnant mothers poses a threat to the health of the naturally non-transmitting mothers should they require nevirapine later as part of their antiretroviral combination therapy. Hence, it is critical to precisely predict mothers who are likely to vertically transmit and offer them nevirapine monotherapy. Accurately predicting pregnant mothers likely to transmit the virus to their infants has long term benefits of saving on drugs and minimizing drug resistance problems. Lack of antibody reactivity to gag p39 antigen during the last trimester of pregnancy could be a predictor of vertical transmission although bigger studies are necessary to verify this observation. The current small sample size could not permit conditional logistic regression analysis to control for variables that could have had an effect on disease progression other than WB band profiles. Hence results should be interpreted cautiously because disease progression and transmission also depend on other factors including host-parasite genetics and interaction.

Control of viral replication following infection has been attributed partly to cytotoxic T lymphocyte (CTL) CD8+ activity. Studies have shown that CTLs directed against g*ag *antigens correlate with improved clinical markers of disease progression [26]. Hence absence of antibody responses to p39 antigen could interfere with normal host neutralization of virus and may contribute to disease progression. Missing bands among the HIV chronically infected women were likely to have been due to diminished antibody responses with progressive disease whilst in recent sero-convertors the missing bands may have been due to immature antibody responses or possibly due to mutations in the *pol *gene [27].

## Conclusion

These data support the rationale of using WB band profiles plus simple laboratory tests like differential counts together with clinical symptoms such as lymphadenopathy in establishing and evaluating disease progression before accessing antiretroviral therapy. This could have important practical applications especially in resource poor settings, where over 95% of the 40 million HIV infected people live, who unfortunately cannot afford costly viral load and CD4 cell tests. However, bigger studies are necessary to shed more light on the use of simple WB band profiles to determine the likelihood of vertical transmission as an initiative to reduce HIV-1 vertical transmission in developing countries.

## Conflict of interests

The authors declare that they have no competing interests.

## Authors' contributions

**KD **collected data and drafted the manuscript, **FM **supervised laboratory analysis, **FZG **collected data, **NEK **collected data, **SR **participated in designing of the study, **MZC **participated in designing of the study, **MPM **performed the statistical analysis and interpretation of results, **BS **participated in designing and coordination of the study. All authors read and corrected the final version of the manuscript.

## Pre-publication history

The pre-publication history for this paper can be accessed here:

http://www.biomedcentral.com/1471-2334/11/7/prepub
